# Nociceptive neurons detect cytokines in arthritis

**DOI:** 10.1186/s13075-014-0470-8

**Published:** 2014-10-30

**Authors:** Hans-Georg Schaible

**Affiliations:** Institute of Physiology I/Neurophysiology, Jena University Hospital - Friedrich Schiller University, Teichgraben 8, D-07740 Jena, Germany

## Abstract

Proinflammatory cytokines are major mediators in the pathogenesis of diseases of joints such as rheumatoid arthritis and osteoarthritis. This review emphasizes that proinflammatory cytokines such as tumor necrosis factor-alpha, interleukin-1beta, interleukin-6 and interleukin-17 are also mediators of pain by directly acting on the nociceptive system. Proportions of nociceptive sensory neurons express receptors for these cytokines, and the application of cytokines rapidly changes the excitability, ion currents and second messenger systems of these neurons. By inducing persistent sensitization of nociceptive sensory neurons (C- and a proportion of Aδ-fibers) for mechanical stimuli in the joint (a process called peripheral sensitization), these cytokines significantly contribute to the persistent hyperalgesia typical for many disease states of the joint. In addition, the disease-associated release of cytokines in the spinal cord supports the generation of central sensitization. The therapeutic neutralization of proinflammatory cytokines thus not only reduces the process of inflammation but may directly reduce hyperalgesia and pain by reversing the neuronal effects of cytokines. It is emerging that different cytokines have different actions on neurons. The neutralization of tumor necrosis factor-alpha reduces both mechanical and thermal hyperalgesia of the joint. The neutralization of interleukin-1beta attenuates thermal hyperalgesia whereas the neutralization of interleukin-6 and interleukin-17 mainly reduces mechanical hyperalgesia. These different effects are partly explained by influencing different target molecules in sensory neurons. For example, in cultured sensory neurons tumor necrosis factor-alpha and interleukin-1beta upregulate the TRPV1 ion channel, which is involved in the transduction of heat stimuli, consistent with an effect of these cytokines in thermal hyperalgesia. By contrast, interleukin-17 upregulates the TRPV4 ion channel, which has a role in the transduction of mechanical stimuli. Thus, the analgesic potential of neutralizing cytokines seems to depend on which cytokine is mainly involved in the particular pain state.

## Introduction

Cytokines are major inflammatory mediators which induce and maintain disease processes such as arthritis. The recognition that cytokines are major players in rheumatoid arthritis (RA) has led to powerful disease-modifying therapies which are based on the neutralization of proinflammatory cytokines such as TNF [1]. Cytokines are also involved in osteoarthritis (OA) [2] and possibly in other joint diseases. The success of neutralization of cytokines in RA and related diseases is documented by the objective attenuation of the disease process as well as by the subjective experience of the patient. For the patient it is most impressive when main symptoms such as pain and inability are significantly improved. This review shows that cytokines play an important role in pain generation. It focuses on the effects of cytokines on peripheral sensory neurons but also alludes to effects of cytokines in the spinal cord.

Arthritic pain has typical features. The patient may experience ongoing pain in the absence of any intentional stimulation. If mechanical stimuli such as movements in the working range and palpation of the joint evoke pain (which is not the case in a healthy joint), the patient is in a state of pathological mechanical hyperalgesia. If normally non-painful warm or cold stimuli evoke pain, the patient experiences thermal hyperalgesia. The basis for hyperalgesia is the sensitization of the nociceptive (pain) system for stimuli, in which the threshold for the excitation of nociceptive neurons (and thus for the elicitation of pain) is lowered and the responses to noxious stimuli are heightened.

**Figure 1:**
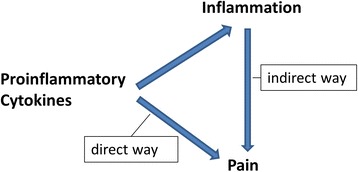
**General diagram of how proinflammatory cytokines contribute to the generation of pain.** The indirect way is induced by mediators such as prostaglandins that are produced during inflammation. The direct way indicates direct effects of cytokines on sensory neurons.

It is usually thought that the neutralization of a proinflammatory cytokine attenuates the disease process, and as a consequence the pain is reduced. Careful observation of experimental models [3,4] and in patients [5] showed, however, that neutralization of a cytokine may reduce the pain quite quickly, well before the attenuation of the disease can be documented. These observations suggest that certain cytokines have a direct role in the generation and maintenance of pain; that is, by targeting the nociceptive system itself. For several proinflammatory cytokines direct effects on nociceptive neurons have been shown: (a) proportions of nociceptive (and other) sensory neurons express receptors for cytokines; (b) in cultured isolated sensory neurons the application of cytokines may activate second messenger systems, change the excitability, modifiy ion currents, and regulate molecules involved in nociception; (c) the injection of some cytokines into normal tissue evokes pain behavior in awake animals and enhances the responsiveness of nociceptive sensory fibers; (d) the neutralization of cytokines may reduce pain well in advance of the attenuation of the inflammatory process. Thus, cytokines contribute to pain indirectly through the generation of inflammation which causes the release of many mediators acting on neurons (for example, prostaglandins) as well as directly by acting on neurons themselves (Figure 1).

Direct effects of cytokines on nociceptive nerve fibers have potentially several important consequences. A pain state may reflect not only the disease process in the joint but also the direct impact of cytokines on the nervous system. In this context it is noteworthy that cytokines are also important mediators of neuropathic pain originating from injury or disease of neurons themselves [6-8]. Thus, hypothetically, cytokines may also induce pathological changes in the nerve fibers in the long term. In fact, many chronic pain states associated with joint diseases show changes in the nervous system which suggest that, in the long term, chronic joint pathology may also trigger some brain pathology [9].

**Figure 2:**
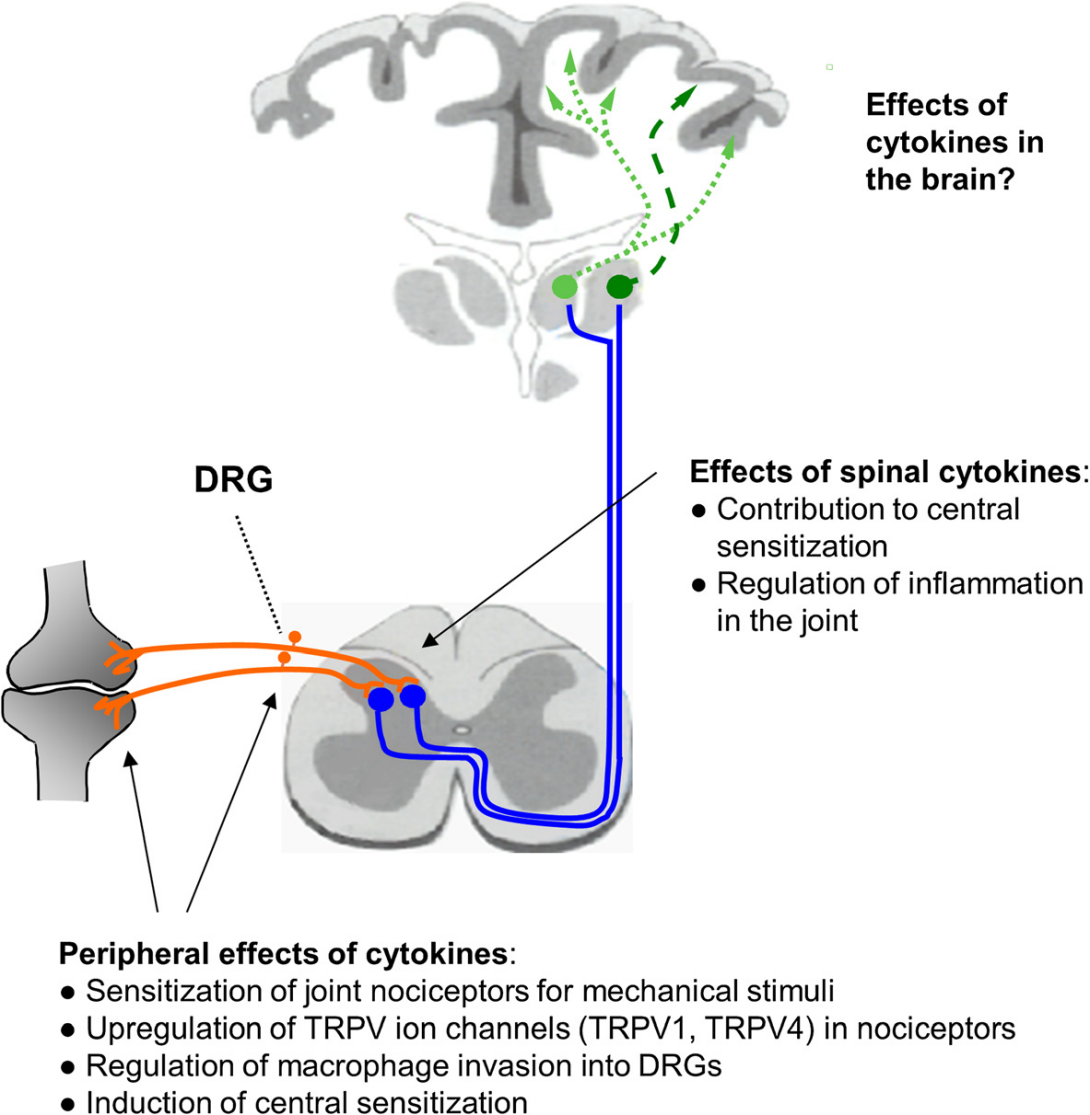
**General overview of effects of peripheral and spinal cytokines on joint nociceptors, the spinal cord, and the brain.** Note that each cytokine has its own profile of actions (see text for details). DRG, dorsal root ganglia; TRP, transient receptor potential.

Nociceptive neurons detect cytokines in the inflamed joint; however, cytokines may have an impact on neurons also at other sites. First, during arthritis macrophages and other inflammatory cells may invade the dorsal root ganglia (DRG) in which the cell bodies of the sensory neurons are located [4,10]. Second, cytokines can be produced and released from glial cells of the spinal cord which may be activated in the course of joint diseases [11]. Thus, the pathology of the joint may create a status in which cytokines within the central nervous system are also involved in the generation of pain and possibly of other symptoms. Finally, spinal cytokines may even influence the peripheral pathology by modifying the efferent neuronal systems which act on peripheral tissues [12,13]. Figure 2 summarizes sites of cytokine actions on neurons and neuronal effects of cytokines.

The following paragraphs concentrate on the proinflammatory cytokines TNF-α, IL-6, IL-1β, and IL-17 because they have been particularly investigated with regard to joint pain. It is likely that other cytokines will be added as soon as their roles in joint pain have been investigated.

## Tumor necrosis factor-α

TNF-α is a major proinflammatory cytokine in RA [1], and may also play a role in OA, which often exhibits marked synovitis [2]. TNF-α also acts on neurons. A proportion of DRG neurons (the cell bodies of sensory neurons) in rat express both TNF receptors (TNFR1 and TNFR2). Some authors identified only TNFR1 in neurons, while localizing TNFR2 in non-neuronal cells in the DRG (reviewed in [14]). In behavioral experiments the injection of TNF-α into healthy tissue induced mechanical and thermal hyperalgesia [15,16].

**Figure 3:**
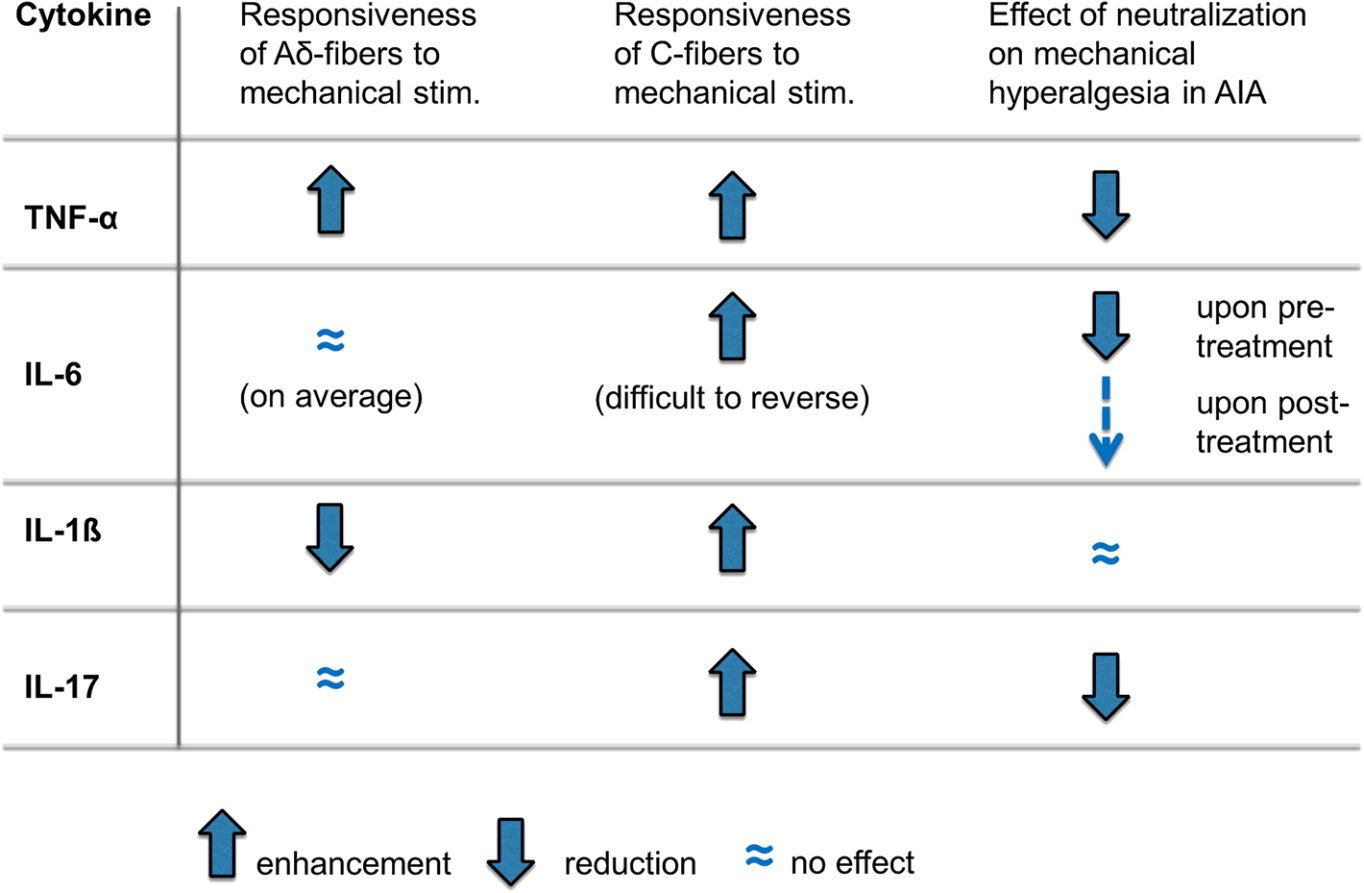
**Effects of proinflammatory cytokines on the responsiveness of nociceptive sensory neurons (Aδ- and C-fibers) of the joint to mechanical stimulation of the joint upon intra-articular injection of different cytokines into the normal knee joint, and long-term effects of the neutralization of these cytokines on pain behavior in the model of antigen-induced arthritis (AIA).**

A single injection of TNF-α into the joint cavity of normal rat knee joints caused a progressive increase of the responses of nociceptive C-fibers (which are unmyelinated) and Aδ-fibers (which are thinly myelinated) to innocuous and noxious rotation of the joint which was dose-dependent and persistent. Thus, TNF-α induced a state of persistent sensitization of joint nociceptors for mechanical stimuli (a basis for mechanical hyperalgesia), similar to inflammation. The effects of TNF-α (and of other cytokines for comparison) on the responsiveness of nociceptive C- and Aδ-fibers to mechanical stimulation of the joint are summarized in Figure 3. The TNF-induced sensitization was prevented by co-administration of the TNF-neutralizing fusion protein etanercept [14]. Although some neuronal TNF-α effects may be indirect and involve other mediators (Figure 1), direct neuronal TNF-α effects are likely because TNF-α causes hyperexcitability in isolated DRG neurons (see below).

The joint is not the only peripheral site at which TNF-α may act on neurons. In the acute phase of joint inflammation DRGs of the segments with inflammation show an invasion of ED1-positive macrophages [4,10]. ED1 is equivalent to human CD68 and widely accepted as a marker for activated macrophages. In DRGs, ED1-positive cells were found near or attached to blood vessels, in close vicinity to neuronal cell bodies and their processes, and between neurons and satellite cells [10]. The invasion was bilateral even if the inflammation (antigen-induced arthritis (AIA)) was only unilateral. Treatment with etanercept or infliximab reduced the macrophage invasion and the expression of vascular cell adhesion molecule-1-like immunoreactivity (which is involved in macrophage migration into the tissue), although the AIA itself was only weakly attenuated by TNF-α neutralization at this time point [10]. Because the cell bodies of DRG neurons express cytokine receptors, macrophages may act on them and cause neuronal effects at this site. Possibly, the macrophage infiltration into the lumbar DRGs of the healthy side may be associated with the mechanical hyperalgesia at the contralateral healthy knee which is observed in the acute stage of AIA [3,10].

**Figure 4:**
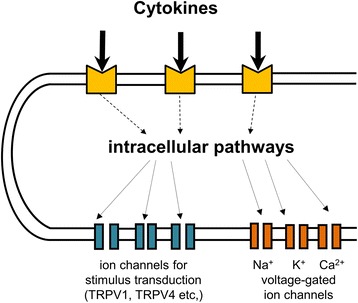
**Model showing how cytokines sensitize nociceptive sensory neurons to stimulation.** The diagram displays the model of a sensory ending of a nociceptive sensory neuron in the joint. The membrane of the neuron expresses ion channels for the transduction of stimuli (their opening by stimuli causes depolarizing sensor potentials in the ending) and voltage-gated ion channels for the regulation of the membrane potential, the excitability and the generation of the action potentials. In addition, the ending expresses receptors for cytokines which activate intracellular pathways. The latter can change the response properties of the ion channels and/or their expression in the membrane. Membrane receptors for other mediators (for example, prostaglandins) are not displayed. TRP, transient receptor potential.

Mechanistically, the sensitization of nociceptive sensory neurons is produced by enhancing the sensitivity and/or the expression of ion channels involved in the transduction of mechanical and thermal stimuli (for example, transient receptor potential (TRP) ion channels), and/or by enhancing the sensitivity of voltage-gated ion channels involved in the general excitability of sensory neurons and the generation of action potentials (Figure 4). The TRPV1 ion channel is opened by heat stimuli, and the elicited ion influx 'converts' the heat stimulus into an electric sensor potential. If TRPV1 is sensitized, it opens at lower temperatures than normal, and this drop of threshold is essential for the generation of thermal hyperalgesia. TRPV4 is a candidate molecule for the transduction of noxious mechanical stimuli, at least under inflammatory conditions (see below), and it is likely to be involved in the generation of mechanical hyperalgesia. The sensitization of voltage-gated Na^+^ channels can enhance the excitability of neurons; that is, less depolarization is required to evoke an action potential. Studies on the effects of mediators on neurons are often performed on isolated and cultured DRG neurons, which allows direct measurement of neuronal effects.

Bath application of TNF-α increased the excitability and voltage-gated (tetrodotoxin-resistant) Na^+^ currents of isolated and cultured DRG neurons within a few minutes, showing a non-genomic effect of TNF-α on ion channels involved in the action potential [14,17]. The increase of Na^+^ currents depends on TNFR1 and the activation of p38 MAP kinase, which possibly phosphorylates Na^+^ channels [17]. The sensitization of joint nociceptors for mechanical stimulation *in vivo* by TNF-α (see above) was also prevented by the p38 inhibitor SB203580, showing the significance of this pathway for mechanical sensitization [14]. The acute application of TNF-α also sensitized TRPV1 ion channels in cutaneous nociceptive neurons, resulting in an enhanced heat-evoked release of the neuropeptide CGRP (calcitonin gene-related peptide) from sensory nerve terminals in rat skin [18]. In cultured DRG neurons long-term incubation with TNF-α for 24 to 48 hours significantly enhanced the proportion of DRG neurons expressing TRPV1, an effect absent in DRG neurons from *tnfr1*^*−/−*^ but not from *tnfr2*^*−/−*^ mice [19]. Thus, TNF-α may further thermal hyperalgesia via upregulation of TRPV1.

*In vivo*, cyclooxygenase inhibitors at a dose of 4 mg/kg completely blocked the TNF-α-induced sensitization of joint nociceptors for mechanical stimuli whereas lower doses allowed TNF-α to sensitize joint afferents. Thus, classical pain therapy at a sufficiently high dose may antagonize TNF-α effects [14].

As well as the peripheral nociceptive system, the spinal cord is important for the generation of clinically relevant pain. During development of inflammation in the joint, nociceptive spinal cord neurons develop a state of hyperexcitability (called central sensitization) which increases the gain of the nociceptive processing [9]. In this context it is important that TNF-α is produced not only in the inflamed tissue but also in the spinal cord, namely by glial cells [11]. Although glial activation is not generally observed in inflammation [20], astroglia and microglia were activated in K/BxN serum transfer arthritis [21] and in models of experimental OA [22]. Both TNF-α in the joint and TNF-α in the spinal cord affect the nociceptive processing in the spinal cord (Figure 2). TNF-α injection into the knee joint cavity increased the responses of spinal cord neurons to mechanical joint stimulation, while intra-articular injection of etanercept attenuated inflammation-evoked spinal activity [23]. These effects can be explained by the modification of the sensory input into the spinal cord because the sensitization of peripheral nerve fibers by TNF-α (see above) will increase the afferent barrage during joint stimulation. However, the spinal application of TNF-α also increased the spinal responses to joint stimulation. Importantly, spinal application of either etanercept or an antibody to TNFR1 during the development of joint inflammation significantly attenuated the generation of inflammation-evoked spinal hyperexcitability, which is characterized by widespread pain sensitization beyond the inflamed joint. These findings indicate that the production and release of TNF-α in the spinal cord supports the generation of inflammation-evoked spinal hypersensitivity [23]. Spinal TNF-α was also implicated in the neuronal control of inflammation in the joint: intrathecal application of TNF inhibitors significantly attenuated joint inflammation [12,13].

From the effects of TNF-α on neurons it should be expected that neutralization of TNF-α reduces pain. In fact, this was observed both in animal models (Figure 3) and in patients with RA. In murine collagen-induced arthritis (CIA) [24], complete Freund's adjuvant (CFA)-induced paw inflammation in rat [4], rat AIA [3], and mouse K/BxN arthritis [21], the neutralization of TNF rapidly reduced inflammatory hyperalgesia in the absence of any other antinociceptive drugs. Typically the antinociceptive effects of TNF neutralization were already observed on the first day of treatment even if swelling was only slightly reduced [3] and gross pathology did not show a difference between treated and untreated animals [3,4]. Similarly, RA patients who received infliximab experienced significant pain reduction after 1 day, although at this early time point the inflammatory process was not reduced [5]. Intra-articular injection of etanercept reduced responses of joint nociceptors to rotation of the inflamed joint within 30 minutes, thus showing that TNF-α neutralization in the joint significantly contributes to the therapeutic effect [3].

TNF-α was also implicated in the generation of neuropathic pain. Damaged nerve fibers are activated by TNF-α released from cells including Schwann cells at the lesion site. TNFR1 is mainly involved in neuropathic pain (reviewed in [25]). TNF-α may also contribute to fibromyalgia because TNF-α application into the normal masseter muscle evoked a long-lasting reduction of the mechanical threshold of muscular Aδ-fibers [26].

## Interleukin-6

IL-6 is a key player in systemic inflammation and arthritis [27]. IL-6-deficient mice show significantly attenuated AIA [28]. In a murine model of human TNF-mediated inflammation, IL-6 was particularly involved in inflammation-evoked osteoclast formation and bone erosion [29]. IL-6 binds either to a membrane-bound IL-6 receptor (IL-6R) or to a soluble IL-6 receptor (sIL-6R), allowing IL-6 trans-signaling onto cells that do not express the membrane receptor. Ultimately the IL-6-IL-6R complex binds to the transmembrane signal-transducing subunit gp130 [30]. The serum, synovial fluid and tissue of RA patients show elevated concentrations of both IL-6 and sIL-6R (reviewed in [31]). While sIL-6R acts as an agonist, circulating soluble gp130 (sgp130) acts as an antagonist, because it binds IL-6-sIL-6R complexes and thus prevents trans-signaling [30]. In murine arthritis models, administration of sgp130 reduced inflammation [28].

DRG neurons express gp130 [32,33]. Injection of IL-6 into rat hindpaw caused behavioral mechanical hyperalgesia [16]. The injection of IL-6 or of IL-6 together with sIL-6R into a normal knee caused a long-lasting sensitization of nociceptive C-fibers for mechanical stimuli applied to the joint whereas the responses of Aδ-fibers remained unaffected on average (Figure 3). The sensitization by IL-6 was prevented by co-administration of sgp130, but notably sgp130 did not reverse the established enhanced mechanosensitivity induced by intra-articular injection of IL6 or IL-6-sIL-6R [34]. Thus, IL-6-induced persistent hyperexcitability is difficult to reverse. Similarly, injection of IL-6 into skeletal muscle enhanced the responses to prostaglandin E2 injected into the muscle for at least 24 hours, showing long-term 'priming' of nociceptive neurons [35].

Knock-out mice lacking gp130 specifically in sensory DRG neurons (SNS-gp130^−/−^) showed reduced inflammatory and tumor-induced pain [36]. Thus, gp130 in afferent neurons is a key regulator of the induction and maintenance of mechanical hypersensitivity [37].

Neurons and glial cells of the spinal cord also express gp130. The membrane receptor IL-6R is mainly found in glial and endothelial cells and sparsely in neurons of the central nervous system (reviewed in [38]). Originally, spinal IL-6 was mainly investigated in the context of neuropathic pain [6,7]; however, rats suffering from articular CFA inflammation and from generalized AIA also showed increased spinal levels of IL-6 (reviewed in [38]).

The application of IL-6-sIL-6R either into the knee joint or topically to the spinal cord increased the responses of spinal neurons to mechanical stimulation of the knee and other parts of the leg, including an expansion of the receptive field size of the neurons, showing the potential of IL-6 to induce central sensitization. Development of knee inflammation evoked significant spinal release of IL-6, and spinal application of sgp130 attenuated the generation of spinal hyperexcitability during development of inflammation. However, spinally applied sgp130 did not reverse established hyperexcitability if inflammation had developed fully [38].

The induction of persistent neuronal hyperexcitability that is difficult to reverse raises the intriguing question of whether neutralization of IL-6 alleviates arthritic pain. In the AIA model a single injection of sgp130 into the knee joint at the time of arthritis induction caused a significant long-term reduction of mechanical hyperalgesia, although acute arthritis *per se* was barely attenuated [31]. By contrast, repeated intraperitoneal injection of sgp130 in the course of AIA reduced mechanical hyperalgesia only weakly, at a time point where AIA is already in the process of remission (Figure 3) [31]. This behavioral pattern of IL-6 neutralization corresponds to the neuronal effects of IL-6, indicating that endogenous IL-6 indeed plays a significant role in the generation of arthritic joint pain but that the IL-6-induced hyperexcitability is difficult to reverse once it is established. These findings suggest, therefore, that IL-6 may play an important role in the chronification and poor reversibility of pain.

A caveat is that sgp130 also regulates the IL-6-related cytokines leukemia inhibiting factor and oncostatin M, but sgp130 has a lower affinity for leukemia inhibiting factor and oncostatin M than for the IL-6-sIL-6R complex, and it prevents mainly trans-signaling by IL-6-sIL-6R complexes [30].

## Interleukin-1β

IL-1β is abundantly expressed in RA [1] and OA [2]. Mice deficient for the naturally occurring inhibitor of IL-1 develop spontaneously erosive arthritis. Neutralization of IL-1β reduced CIA but not adjuvant arthritis and AIA. Bone loss was reduced in CIA and adjuvant arthritis (reviewed in [39]). Anakinra, an IL-1 receptor (IL-1R1) antagonist, had only limited efficacy in human RA [1].

IL-1β binds to the cell surface receptors IL-1RI and IL-1RII. IL-1RI transduces the biological signal of IL-1β into cells and IL-1RII serves as a decoy receptor. Sensory neurons express only IL-1RI [40,41]. In rats 26.4 ± 2.9% of all DRG neurons of small and medium size expressed IL-1R1 but, interestingly, during immunization in the AIA model the proportion increased up to 60% of DRG neurons and persisted during subsequent AIA [39]. IL-1β contributes to pain and hyperalgesia (reviewed in [42]). In rat, intraplantar injection of IL-1β induced cutaneous hyperalgesia and transient ongoing discharges [43].

Recordings from joint afferents showed that IL-1β sensitizes nociceptive C-fibers of the joint to mechanical stimuli. Interestingly, however, the sensitivity of nociceptive Aδ-fibers was simultaneously significantly reduced by IL-1β (Figure 3). The reason for the latter effect is unknown. Because Aδ- and C-fibers converge onto the same spinal cord neurons, the increase of the C-fiber input into the spinal cord is at least partly compensated for by the decrease of the Aδ-fiber input after IL-1β [39].

In isolated DRG neurons, IL-1β increased excitability via p38 MAP kinase and enhanced tetrodotoxin-resistant Na^+^ currents [44], suppressed voltage-gated K^+^ channels [45], and increased TRPV1 currents [41]. The exposure of DRG neurons to 1 nmol/l (but not to 10 nmol/l) IL-1β significantly upregulated the proportion of DRG neurons expressing the TRPV1 channel (prevented by the IL-1 receptor antagonist IL-1Ra) [39]. In addition, IL-1β changes the activity of the enzyme G-protein-coupled receptor kinase 2 (GRK2). GRK2 regulates the responsiveness of multiple G-protein-coupled receptors, and downregulation of GRK2 by IL-1β reduces the internalization of these receptors and thus promotes nociception [46].

Because the pattern of effects of IL-1β on sensory neurons of the joint (opposite effects on C- and Aδ-fibers) is different to that of TNF-α and IL-6, it is an intriguing question how neutralization of IL-1β affects pain in arthritis. In the AIA model, treatment with anakinra had only marginal effects on the severity of arthritis and mechanical hyperalgesia, which may result from the opposite effects of IL-1β on C- and Aδ-fibers [39] (Figure 3). But anakinra consistently and significantly reduced the thermal hyperalgesia, along with a reduction of the expression of TRPV1 in DRGs during AIA [39]. Because the major burden of RA is mechanical hyperalgesia and not thermal hyperalgesia, anakinra may not cause pain relief in RA. By contrast, in gout, a 'hot' inflammation, thermal hyperalgesia may be more important, and in gout neutralization of IL-1β is analgesic [47].

## Interleukin-17

Recently, IL-17 became the focus of research because it was identified as a major mediator of immunity and inflammation, such as in RA, multiple sclerosis and other disorders [1,48]. IL-17A, the prototype member, induces the production of mediators of innate and adaptive immunity [48,49]. The synovial fluid of RA patients exhibits elevated IL-17A levels [48]. Reduction of IL-17A ameliorated disease activities in preclinical models of RA whereas overexpression of IL-17A aggravated CIA [48]. The use of monoclonal antibodies against IL-17 in humans effectively reduced inflammatory diseases [49,50].

The receptor IL-17RA is ubiquitously expressed. In DRG sections and cultured DRG neurons the majority of neurons show cytoplasmatic expression of IL-17RA. An antibody directed to the amino-terminal extracellular domain of IL-17RA labeled 43 ± 4% of cultured DRG neurons, which were mainly small and medium sized [51], suggesting that IL-17A in the extracellular fluid affects a proportion of sensory neurons.

Behaviorally, the intra-articular injection of IL-17 caused experimental hyperalgesia [52]. Wild-type and *Il17a*^−/−^ mice showed similar nociceptive mechanical and thermal thresholds in the absence of inflammation but after induction of zymosan-induced paw inflammation (zymosan activates lymphoid tissue inducer-like cells as an innate source of IL-17 and IL-22) the mechanical threshold was less reduced in *Il17a*^−/−^ than in wild-type mice, indicating that IL-17A contributes to mechanical hyperalgesia even when paw swelling was identical in *Il17a*^−/−^ and wild-type mice. Wild-type and *Il17a*^−/−^ mice did not differ in the development of thermal hyperalgesia [51], except at a later stage at which IL-17A may also activate inflammatory cascades which change thermal sensitivity [52,53].

A single injection of IL-17A into the normal knee joint caused a dose-dependent and slowly developing and persistent increase of the responses of nociceptive C-fibers to both innocuous and noxious rotation of the joint [54] (doses were similar to those used in behavioral experiments in mice [52,53]). The IL-17A-induced sensitization was prevented by neither etanercept nor sgp130; thus, IL-17A itself sensitizes joint afferents [54]. Interestingly, nociceptive Aδ-fibers were only sensitized at a deteriorating dose for C-fibers, suggesting that IL-17A mainly sensitizes C-fibers (Figure 3). At a very low dose, IL-17A reduced the responses of Aδ-fibers and may therefore reduce the input from the periphery [54].

Cellular studies support neuronal effects of IL-17. In isolated and cultured rat DRG neurons, IL-17A evoked phosphorylation of protein kinase B (PKB/Akt) and extracellular-regulated kinase (ERK) within 5 minutes. IL-17A generated hyperexcitability of small to medium-sized DRG neurons within 5 minutes after bath application, showing regulation of voltage-gated ion channels [54]. After long-term exposure to IL-17A more DRG neurons exhibited pERK [51], a mechanism possibly involved in long-term hyperexcitability.

Incubation of DRG neurons with IL-17A did not upregulate TRPV1 (consistent with a lack of IL-17 on heat sensitivity); however, IL-17A upregulated TRPV4. The TRPV4 ion channel is gated by temperature in the innocuous range with a maximum at 37°C, but is also considered a candidate molecule for the transduction of noxious mechanical stimuli, at least under inflammatory conditions [55]. Although TRPV4 is not the only TRP channel involved in mechanical hyperalgesia, the upregulation of TRPV4 by IL-17A in conjunction with the induction of mechanical hyperexcitability by IL-17A reinforces a functional role of TRPV4 in the state of mechanical hyperalgesia.

In the AIA mouse model an antibody against IL-17 slightly reduced the swelling, but significantly reduced the guarding score and secondary mechanical hyperalgesia at the paws [54], thus further supporting a role of IL-17A in mechanonociception (Figure 3). In human RA, a fully human antibody (AIN457) to IL-17A reduced inflammation and significantly decreased the number of joints with pressure pain as early as 1 week and up to 16 weeks after a single or two injections of the antibody [50], showing a role for IL-17 in human mechanical hyperalgesia.

Since IL-17RA is also expressed in satellite cells of DRGs, IL-17A may also be involved in neuropathic pain because satellite cells play a role in the pathological destructive and repair processes in DRGs in the aftermath of neuronal damage [56].

## Conclusion

**Figure 5:**
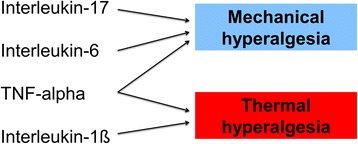
**Profile of different cytokines in the generation of hyperalgesia.** IL-17, IL-6, and TNF-α cause mechanical hyperalgesia, whereas thermal hyperalgesia is mainly induced by TNF-α and IL-1β.

The data show that cytokines are not only mediators of disease processes but are also pain mediators by directly acting on the nociceptive system. Through comparison of the effects of different cytokines on sensory neurons the concept begins to emerge that different cytokines are involved in particular qualities of pain, such as mechanical or thermal hyperalgesia (Figure 5). The specific effects of particular cytokines result from their different effects on Aδ- and C-fibers and from their different target molecules of nociception (for example, TRPV1 versus TRPV4). This conclusion is substantially supported by the treatment studies using the AIA model, which show that neutralization of different cytokines reduced the particular quality of hyperalgesia that is generated by a particular cytokine. Furthermore, it is becoming evident from both the effects of cytokines on nerve fibers and the treatment studies that the neuronal effects of some cytokines (for example, TNF-α) can be more easily reversed than the effects of others (for example, IL-6), suggesting that different cytokines have a different potential to induce chronic pain states. Available clinical data also support this concept (for example, the rapid reduction of mechanical hyperalgesia by neutralization of TNF-α in animal models of arthritis and in RA patients).

As an extension of this concept it may be speculated that the pain pattern in a patient and the likelihood to reduce the pain by cytokine neutralization depends on the cytokines which are predominant in the pathogenesis of the disease. If TNF-α is mainly involved, the chance may be high to achieve pain reduction with neutralization of TNF-α because TNF-α effects are reversible. By contrast, if IL-6 is predominant, the chance of pain reduction may be much smaller because IL-6-induced sensitization is more difficult to reverse. If the inflammation is hot (for example, during acute gout), the neutralization of IL-1β may cause pain reduction because TRPV1 is involved whereas this treatment will not reduce the mechanical hyperalgesia in chronic RA. Careful monitoring of the effects of neutralization of cytokines on pain in the clinical setting may help to support or reject this concept.

Further challenges for research are to expand our knowledge about the role of the cytokine-induced neuronal activation in the (reflex) control of inflammation [12,13]. Furthermore, it has to be explored whether the reduction of brain activity by cytokine neutralization [5] is based on the neutralization of peripheral cytokine effects or whether cytokines in the brain may also be involved in pain generation. For example, TNF-α has a significant impact on synaptic functions in brain regions such as the hippocampus [57], and it is possible, therefore, that changes in TNF-α are partly responsible for symptoms associated with chronic inflammation.

## Note

This article is part of the series ‘*At the interface between immunology and neurology in rheumatic diseases’*, edited by Rainer Straub. Other articles in this series can be found at http://arthritis-research.com/series/neurology.

## Abbreviations

AIA: antigen-induced arthritis
CFA: complete Freund's adjuvant
CIA: collagen-induced arthritis
DRG: dorsal root ganglia
GRK2: G-protein-coupled receptor kinase 2
IL: interleukin
IL-6R: IL-6 receptor
OA: osteoarthritis
RA: rheumatoid arthritis
sgp130: soluble gp130
sIL-6R: soluble IL-6 receptor
TNF: tumor necrosis factor
TNFR: tumor necrosis factor receptor
TRP: transient receptor potential
